# Commentary on ‘Outcome comparison of meniscal allograft transplantation (MAT) and meniscal scaffold implantation (MSI): a systematic review’

**DOI:** 10.1097/JS9.0000000000001703

**Published:** 2024-05-29

**Authors:** Yeabsira W. Atena, Mahalaqua N. Khatib, Quazi S. Zahiruddin, Sarvesh Rustagi, Prakasini Satapathy, Ayush Anand

**Affiliations:** a Myungsung Medical College, Addis Ababa, Ethiopia; b Division of Evidence Synthesis; c South Asia Infant Feeding Research Network (SAIFRN), Division of Evidence Synthesis, Global Consortium of Public Health and Research, Datta Meghe Institute of Higher Education, Wardha; d School of Applied and Life Sciences, Uttaranchal University, Dehradun, Uttarakhand; e Center for Global Health Research, Saveetha Medical College and Hospital, Saveetha Institute of Medical and Technical Sciences, Saveetha University, Chennai, India; f Medical Laboratories Techniques Department, Al-Mustaqbal University, Hillah, Babil, Iraq; g BP Koirala Institute of Health Sciences, Dharan, Nepal; h MediSurg Research, Darbhanga, India

*Dear Editor,*

Meniscal injuries pose a significant challenge in orthopedic surgery, particularly given the crucial role of the meniscus in knee joint function. The advent of Meniscal Allograft Transplantation (MAT) and Meniscal Scaffold Implantation (MSI) has provided promising solutions for patients with severe meniscal damage. The recent systematic review by Dong *et al*.^1^ offers a comprehensive comparison of these two techniques, shedding light on their respective outcomes and potential for surgical application.

The systematic review by Dong *et al*. is a robust analysis encompassing 83 studies and 3932 patients with Lysholm score as the primary outcome measure, making it one of the most extensive reviews on the subject to date. Secondary outcomes included Patient-Reported Outcome Measures, Return to Sports rates, survival rates, and complication rates. Both MAT and MSI resulted in significant improvements in the Lysholm score, indicating enhanced knee function post-surgery. MSI showed superior preoperative and postoperative Lysholm scores compared to MAT, although the mean improvements were similar between the two groups. Specifically, MSI demonstrated higher improvements in IKDC, (KOOS) symptom, KOOS pain, and KOOS activities of daily living scores compared to MAT. However, MAT patients experienced better pain relief as indicated by the visual analog scale pain scale. Also, MSI exhibited a higher 10-year survival rate (81.85%) compared to MAT (66.57%), highlighting its potential for longer-term efficacy. Moreover, the overall complication rate was significantly lower in MSI (9.64%) compared to MAT (17.52%).

The findings of this review have several important implications for surgical practice. First, the superior long-term survival and lower complication rates associated with MSI suggest it should be considered preferentially for patients with partial meniscal defects. This is particularly relevant for older patients or those with a history of meniscectomy. Second, given the technical complexities and potential for graft extrusion and immunologic rejection associated with MAT, MSI offers a less invasive alternative with a more straightforward implantation process. Third, surgeons should be aware of the differences in postoperative pain relief and functional improvements between the two techniques. Tailored rehabilitation protocols may be necessary to maximize the benefits of each approach.

While the systematic review provides valuable insights, it also has several limitations. First, the inclusion of various study designs (randomized controlled trials, cohort studies, case series) introduces heterogeneity, which may affect the comparability of results. Second, only English-language studies were included, potentially omitting relevant data from non-English sources. Third, differences in patient demographics, surgical techniques, and follow-up durations were not fully addressed, which could influence the outcomes.

To build on the findings of this review and address its limitations, future research should focus on the following areas. First, more high-quality, long-term studies are needed to evaluate the durability and long-term clinical outcomes of MSI, particularly for different scaffold materials like Actifit and CMI. Second, well-designed randomized controlled trials comparing MAT and MSI directly, with standardized outcome measures and follow-up protocols, are essential to establish clearer comparative effectiveness. Third, further research into the biomechanical properties of meniscal scaffolds versus allografts will help in understanding their load-bearing capacities and functional integration within the knee joint. Fourth, economic evaluations comparing the cost-effectiveness of MAT and MSI will provide valuable information for healthcare decision-makers and policymakers.

In conclusion, the systematic review by Dong *et al*. represents a significant contribution to the field of knee surgery, offering a comprehensive comparison of MAT and MSI. Both techniques demonstrate efficacy in improving knee function, with MSI showing advantages in long-term survival and lower complication rates. These findings underscore the importance of patient-specific treatment planning and highlight the need for continued research to optimize meniscal repair strategies. As we advance, integrating clinical outcomes with biomechanical insights and cost-effectiveness analyses will be crucial in refining our approach to meniscal injuries.

## Ethical approval

Not applicable.

## Consent

Not applicable.

## Source of funding

Not applicable.

## Author contribution

Y.W.A.: conceptualization, project administration, supervision, validation, writing – original draft, and writing – review and editing; M.N.K., Q.S.Z., S.R., and P.S.: writing – original draft, and writing – review and editing; A.A.: supervision, validation, and writing – review and editing.

## Conflicts of interest disclosure

There are no conflicts of interest.

## Research registration unique identifying number (UIN)

Not applicable.

## Guarantor

Yeabsira W. Atena.

## Data availability statement

Not applicable.

## Provenance and peer review

Not commissioned, externally peer-reviewed.

## Presentation

Not applicable.

## Assistance with the study

Not applicable.
